# The activation, clinical course, and clinical outcome of using an unconventional electrode configuration in a patient newly implanted with Inspire® therapy: a case report

**DOI:** 10.3389/fmed.2025.1501242

**Published:** 2025-02-27

**Authors:** Ruchir P. Patel, Chelsie E. Rohrscheib

**Affiliations:** ^1^The Insomnia and Sleep Institute of Arizona, Scottsdale, AZ, United States; ^2^Wesper, Inc., New York, NY, United States

**Keywords:** obstructive sleep apnea, sleep disorders, hypoglossal nerve stimulation, Inspire®, hypoxic burden, CPAP intolerance, care pathways

## Abstract

Hypoglossal nerve stimulation therapy via the Inspire® implant is a common alternative to positive airway pressure (PAP) treatment for obstructive sleep apnea (OSA). While hypoglossal nerve stimulation (HGNS) therapy offers a high rate of successful treatment outcomes, the post-activation care pathway, which involves gradual titration of the amplitude to achieve both subjective and objective improvements, can be lengthy, ranging from 3 to 12 months post-activation. Here, we report a case of a 55-year-old man with severe obstructive sleep apnea and a history of hypertension who underwent successful activation and titration of the Inspire® implant to achieve subjective and objective relief within 8 weeks post-implantation and 5 weeks post-activation, using an unconventional starting electrode configuration. This case highlights the need for further exploration of alternative Inspire® activation and management protocols that may lead to improved patient outcomes and higher success rates.

## Introduction

The Inspire® hypoglossal nerve stimulation (HGNS) device by Inspire Medical Systems Inc. is a common treatment for patients with moderate to severe obstructive sleep apnea (OSA). This device improves OSA by stimulating the hypoglossal nerve (HN), which controls the muscles of the tongue, preventing it from blocking the airway (1). The use of Inspire® as an alternative to positive airway pressure (PAP) therapy has seen substantial growth in recent years, with over 70,000 patients treated as of 2023 (2). While research supports the potential for excellent outcomes with Inspire®, the post-activation care pathway involving gradual titration of the amplitude can be lengthy, ranging from 3 to 12 months (3). This time-consuming process often leads to patient frustration and dissatisfaction. Furthermore, frustration among physicians can result in a lack of consideration of Inspire® as a viable treatment option for their patients.

**Figure 1:**
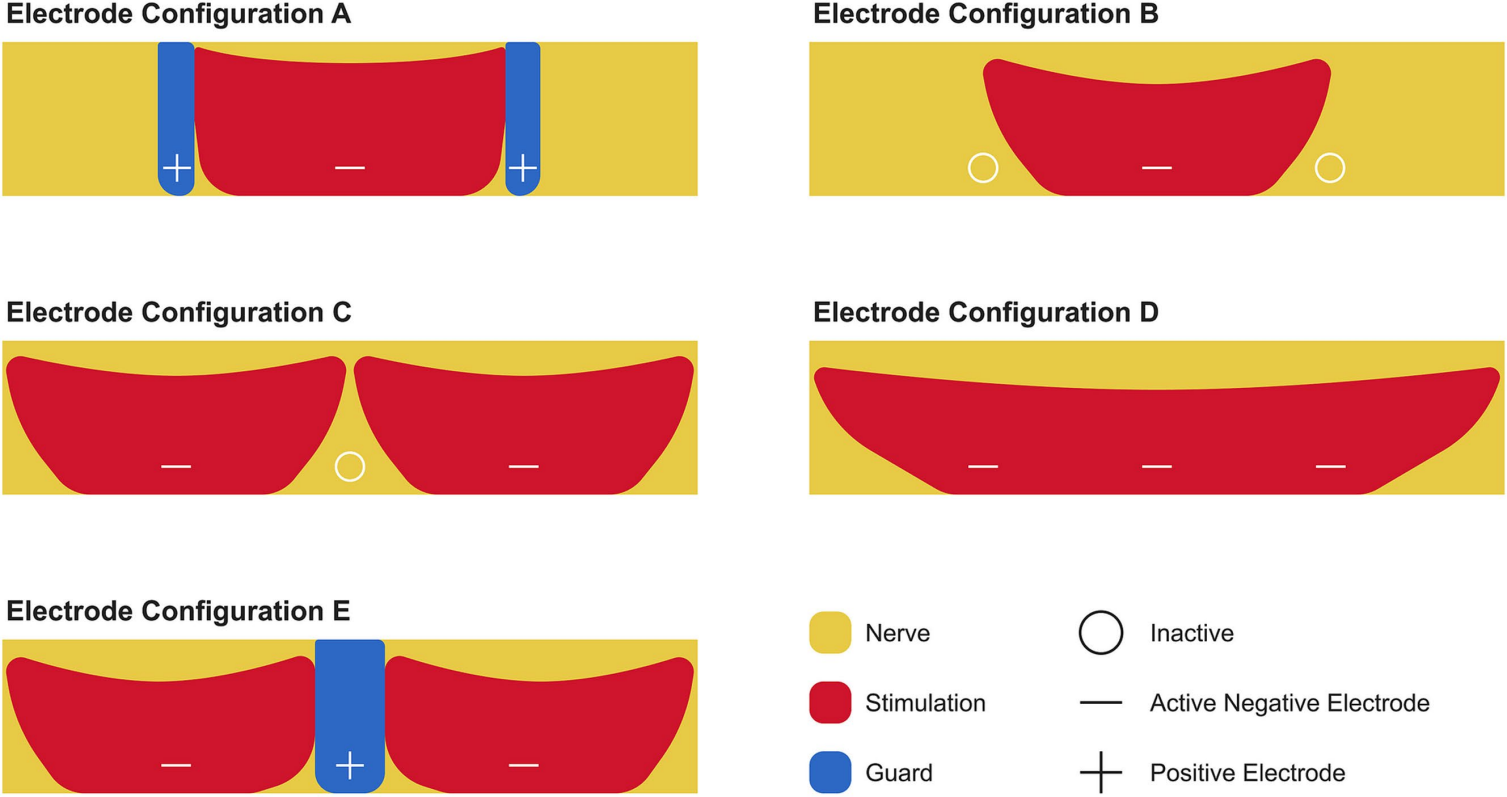
Electrode configurations for stimulating the hypoglossal nerve using the Inspire® device. The distal branch of the hypoglossal nerve (yellow) is stimulated via the cuff, which contains a system of three electrodes. These electrodes can be programmed to function as a positive (+) or negative (−) electrode or switched off (o). The positive electrode acts as a guard (blue) that limits the area of stimulation (red). The recommended electrode configuration by Inspire Medical Systems Inc. is Electrode A.

The Inspire® device consists of several key components, including an implantable pulse generator, a stimulation lead, a respiratory sensing lead, and an external remote control. The single-cuff stimulation lead can be configured to operate in five different electrode configurations (Figure 1). Inspire Medical Systems Inc. refers to these configurations as Electrode A (+ − +), a bipolar configuration with a guarded cathode; Electrode B (o – o), an unipolar configuration with a single centered cathode; Electrode C (− o –), an unipolar configuration with dual outer cathodes; Electrode D (− − –), a grouped cathode (broad unipolar); and Electrode E (− + −), a bipolar configuration with dual outer cathodes and a center guard (3). Each can be strategically positioned along the length of the distal branch of the HN to stimulate the nerve in synchronization with the patient’s breathing, resulting in tongue protrusion, which prevents airway obstruction (4, 5).

Based on the STAR trial published in 2014, Inspire Medical Systems Inc. chose the electrode configuration known as Electrode A, or plus-minus-plus (+ − +), as the standard default electrode configuration to activate all patients (6). Electrode A applies stimulation to the distal branch of the HN through the middle electrode, which serves as the anode, while the two outer electrodes serve as the cathodes. To date, Inspire Medical Systems Inc. has not explored or recommended using alternative electrode configurations as a means to improve outcomes in patients who do not respond to (+ − +) or to reduce the length of titration time.

This case study outlines a novel Inspire® activation approach using the unipolar electrode configuration of Electrode B, or off-minus-off (o – o), in which the middle electrode is utilized to deliver stimulation over a broader area across the distal branch of the HN. The use of this electrode configuration allows for a wider energy field to be applied across the distal branch of the HN, thereby resulting in a different effect on nerve fiber recruitment, muscle activation, and consequently, tongue movement.

## Case description

A 55-year-old man with a medical history significant for hypertension and severe OSA [the Apnea–Hypopnea Index (AHI) ≥30] was presented for Inspire® therapy due to difficulty tolerating PAP therapy. His OSA was re-evaluated over two nights using Wesper’s home sleep apnea test (HSAT) in August 2023 (7). During the first night, his AHI was 28.6 with an HSAT-O2 nadir value of 76%. On the second night, his AHI was 30.9 with an O2 nadir value of 75%.

**Figure 2:**
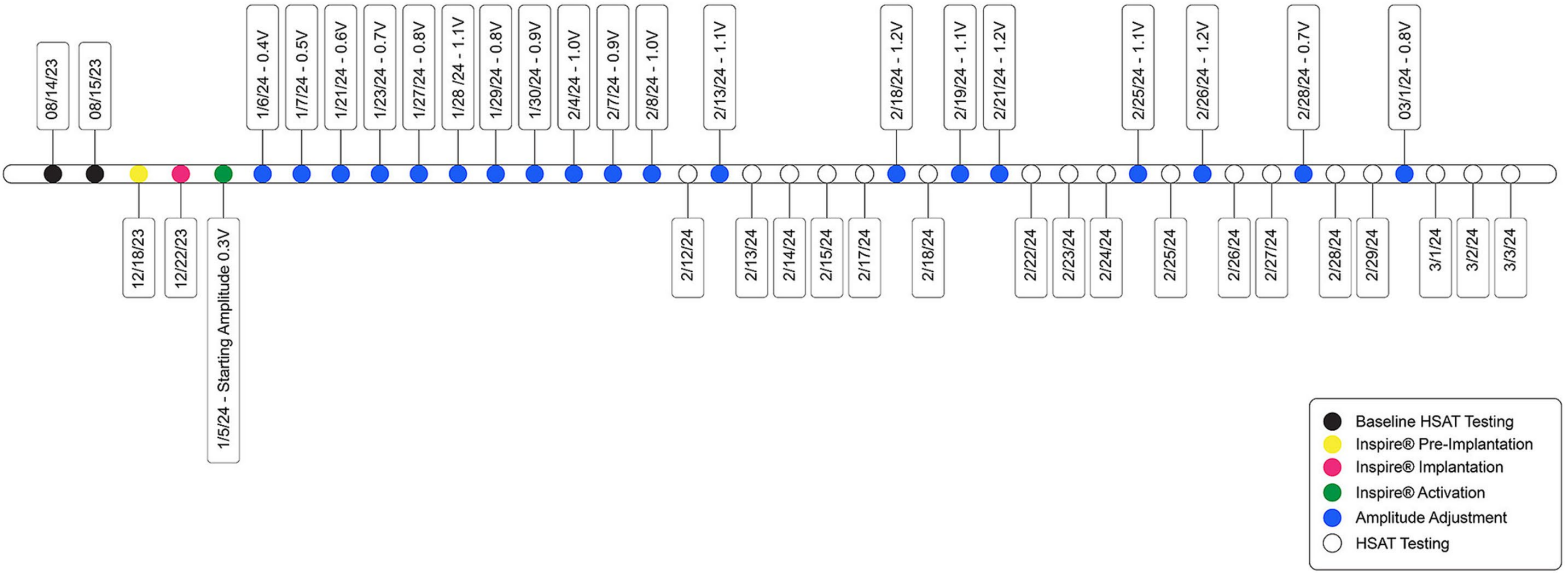
Timeline of patient care, from Inspire® implantation through activation and amplitude titration.

Inspire® was implanted on 22 December 2023 and activated 3 weeks later (Figure 2). The patient agreed to participate in a novel activation approach using Electrode B (o – o) (Table 1). In this configuration, the electrode labeled “o” is inactive, meaning no electrical stimulation is delivered, whereas the “–” negative electrode is active, serving as a cathode and generating electrical current to stimulate the HN. Initially, before activating, programming, and titrating Electrode B (o – o), the patient underwent the standard activation protocol with Electrode A (+ − +) for the documentation of these settings. Electrode B was selected due to its more powerful unipolar configuration, which allows for effective midline protrusion of the tongue at lower amplitudes. The primary advantage of Electrode B lies in the way the energy field is applied to the nerve, leading to a distinct muscle recruitment pattern of the genioglossus and geniohyoid muscles. This often results in a more centralized activation, generating symmetric forward traction on the tongue base and achieving greater airway opening. In addition, the lower amplitude required with this configuration enhances patient comfort while achieving superior airway patency, compared to Electrode A. As per Inspire Medical Systems Inc., there are no known contraindications for Electrode B.

**Table 1:** Inspire® activation parameters.

Initial activation parameters		
	Electrode A (+ − +)	Electrode B (o – o)
Sensational threshold	0.6 V	0.2 V
Functional threshold	0.9 V	0.3 V
Tongue movement	Symmetric midline protrusion	

Initial programming		
Electrode configuration	(o – o)	
Amplitude configuration	0.2 V – 1.2 V	
Starting amplitude	0.3 V	
Rate	40 Hz	
Pulse width	60 μs	
Start delay	30 min	
Pause delay	15 min	
Total therapy duration	8 h	

Novel initial Inspire® activation protocol for Electrode A (+ − +) and Electrode B (o – o), along with initial programming for the novel Electrode B configuration setting. The patient initially underwent the standard activation protocol with Electrode A (+ − +) to document these settings before activating, programming, and titrating Electrode B (o – o).

The patient was advised to increase the amplitude at a frequency of every fifth to seventh night, depending on tolerance. If an amplitude level was uncomfortable, he was instructed to decrease it by one level until his next clinic visit. At the 2-week follow-up visit, the patient was titrated to an amplitude of 0.8 V and reported feeling comfortable with the stimulation. He noted that he was sleeping 90–120 min longer per night compared to before starting Inspire® therapy, as well as a reduction in daytime sleepiness.

The patient was seen 2 weeks after titrating to an amplitude of 1.0 V and was doing well, but he felt the stimulation was too strong. Therefore, the rate was reduced to 30 Hz, and the amplitude was increased to 1.1 V. At the subsequent 2-week follow-up, the patient reported that an amplitude of 1.2 V began disrupting his sleep, so he remained at 1.1 V. The pulse width was then increased to 120 μs, and the rate was increased to 33 Hz, while the amplitude was reduced to 0.7 V. In these settings, the stimulation felt comfortable, and the patient continued to maintain good tongue protrusion.

**Figure 3:**
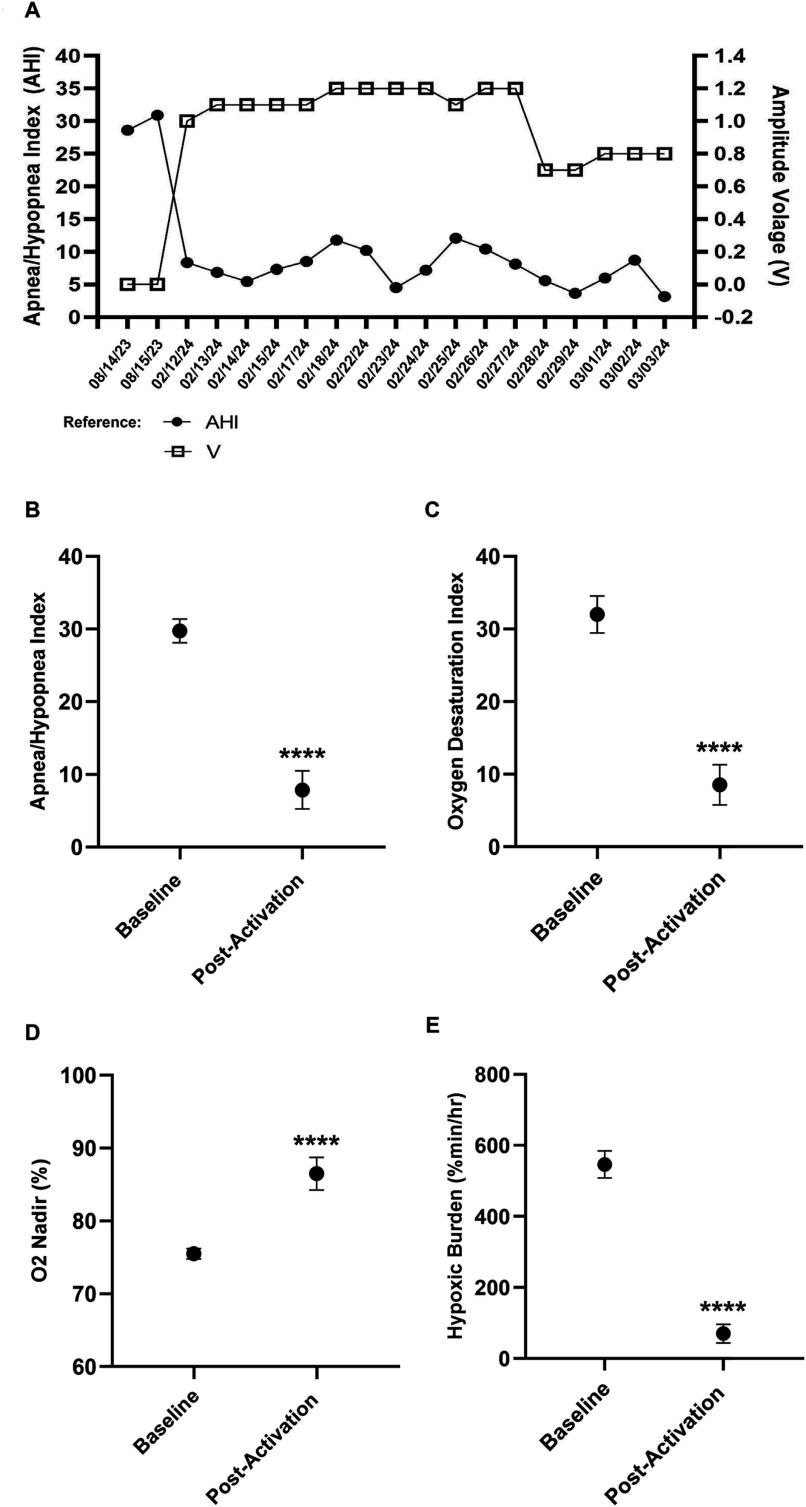
Improvements in key obstructive sleep apnea indices after Inspire® activation. Baseline data represent pre-activation. **(A)** Pre-activation and postactivation Apnea-Hypopnea Index (AHI) versus amplitude. **(B)** Pre-activation and post-activation AHI. **(C)** Pre-activation and post-activation oxygen desaturation index. **(D)** Pre-activation and post-activation minimum SpO2. **(E)** Pre-activation and post-activation hypoxic burden.

During the titration process, the patient’s OSA was re-evaluated using Wesper’s HSAT (*n* = 17) after each amplitude voltage adjustment. These results were compared to the patient’s pre-implantation (*n* = 2) results (Figures 3A–E). Significance was determined using a *t*-test. The patient’s AHI, oxygen desaturation index (ODI), O2 nadir, and hypoxic burden (HB) were assessed to evaluate improvements in OSA. The patient’s average post-activation AHI [Baseline AHI: 29.7, SD = 1.62; Post-activation AHI: 7.5, SD = 2.64; *t*(17) = 11.44; *p* < 0.0001; Figure 3A], ODI [Baseline ODI: 32.0; SD = 2.54; Post-activation ODI: 8.29; SD = 2.74; *t*(17) = 11.60; *p* < 0.0001; Figure 3B], O2 nadir [Baseline nadir: 75.5%; SD = 0.70; Post-activation nadir: 86.8%; SD = 2.27; *t*(17) = 6.85; *p* < 0.0001; Figure 3C] and HB [Baseline HB: 546.58%min/h; SD = 38.0; Post-activation HB: 67.65%min/h; SD = 25.66; *t*(17) = 24.13; *p* < 0.0001; Figure 3D] showed significant improvements with the (o – o) electrode configuration.

The testing process determined that his AHI improvement at the final amplitude of 0.8 V was not significantly different compared to higher amplitudes of ≥1.0 V (Figure 3A), further confirming that 0.8 V was the ideal amplitude setting for both objective and subjective improvements. His most recent HSAT result on 3rd March 2024 revealed a residual AHI value of 3.16, ODI value of 4.43, O2 nadir value of 90%, and HB value of 34.6%min/h. The titration process was completed 8 weeks after the implantation and 5 weeks after the activation compared to the average recommended completion time of 12–17 weeks.

The patient presented for his final follow-up visit 1 week later, reporting that he was sleeping very well at an amplitude of 0.8 V and no longer noticing the stimulation after the adjustments made to the stimulation parameters the previous week. His most recent HSAT result revealed a residual AHI value of 3.2 and an O2 nadir value of 90%. In addition, his score on the Epworth Sleepiness Scale decreased from 10 at baseline to 6 post-treatment, and his Insomnia Severity Index score decreased from 10 to 6. The titration process was completed 8 weeks after the implantation and 5 weeks after the activation, compared to the average recommended completion time of 12–17 weeks.

## Discussion

The Inspire® implant was approved by the FDA in 2014 and has since become a powerful alternative therapeutic option for patients with moderate to severe OSA. While patient selection is of great importance to ensure treatment appropriateness and optimize outcomes, it is also understood that even the most optimal patient may not achieve both subjective and objective improvements with the recommended default electrode configuration within the estimated 90-day titration timeframe. This case successfully demonstrates that alternative Inspire® electrode configurations can be used to achieve both subjective and objective improvements in obstructive sleep apnea (OSA), as well as reduce titration time. A limitation of this study is that electrode configurations A, C, D, and E were not tested, which may limit the generalizability of the findings and highlights the need for further research involving a broader range of configurations.

There are multiple individual factors that may result in an individual not having a successful subjective and/or objective outcome with Inspire® therapy. Common reasons include comorbid conditions such as generalized anxiety disorder, chronic insomnia, restless legs syndrome, a low arousal threshold, or chronic pain syndrome. The patient’s beliefs and behaviors may also affect the outcomes. These include unrealistic expectations, difficulty operating the remote control correctly, a lack of understanding of the self-titration protocol, or even fatigue during the lengthy titration period.

Many patients are unable to be successfully titrated to an effective amplitude within the initial 90-day post-activation period to achieve subjective and/or objective improvement. This can lead to patient dissatisfaction, distrust of the therapy, and a loss of confidence due to the lack of a successful treatment outcome after such a lengthy titration process. The process of initiating Inspire® therapy is often described as a journey rather than a sprint, but even a journey has its limit. As a result, protocols must be evaluated and re-evaluated to determine if there is a more effective and efficient method that can achieve the same or even better outcomes, ultimately improving patient satisfaction and even increasing physician acceptance of this therapeutic alternative.

To improve patient satisfaction, physician satisfaction, clinical workflows, and patient outcomes, more streamlined protocols need to be established utilizing alternative electrode configurations. It is well established that the different electrode configurations currently available for the Inspire® implant produce unique patterns of neurostimulation. These patterns may influence the patient’s tolerance of the therapy and the movement of the tongue. Thus, alternative configurations have unique effects on nerve fiber recruitment and the activation of the genioglossus and geniohyoid muscles, resulting in varying patterns of airway dilatation (4, 8).

Furthermore, the availability of longitudinal efficacy data during the titration phase of hypoglossal nerve stimulation therapy enables clinicians to make faster and more informed management decisions. The current model of care does not allow for the regular collection of efficacy data as minor or major programming adjustments are being made. Access to this type of data could potentially shorten the titration period and also provide the option of alternative electrode configurations for initiating the therapy, rather than recommending only a single electrode configuration.

In conclusion, this case report demonstrates the potential of alternative Inspire® electrode configurations to improve both subjective and objective outcomes in patients with obstructive sleep apnea (OSA). The use of the unipolar Electrode B configuration resulted in significant improvements in the AHI, oxygen saturation, and overall sleep quality, while also shortening the titration period compared to standard practices. However, a limitation of this study is that not all electrode configurations were tested, which may limit the generalizability of the findings. Further research is needed to explore a broader range of electrode configurations and develop more efficient activation protocols to improve patient outcomes, reduce titration time, and enhance both patient and physician satisfaction with Inspire® therapy.

## Data Availability

The datasets presented in this study can be found in online repositories. The names of the repository/repositories and accession number(s) can be found below: https://doi.org/10.6084/m9.figshare.26870662.v1.
